# A Digital Tool to Promote Alcohol and Drug Use Screening, Brief Intervention, and Referral to Treatment Skill Translation: A Mobile App Development and Randomized Controlled Trial Protocol

**DOI:** 10.2196/resprot.7070

**Published:** 2017-04-18

**Authors:** Derek D Satre, Khanh Ly, Maria Wamsley, Alexa Curtis, Jason Satterfield

**Affiliations:** ^1^ Weill Institute for Neurosciences Department of Psychiatry University of California San Francisco, CA United States; ^2^ Kaiser Permanente Northern California Division of Research Oakland, CA United States; ^3^ Division of General Internal Medicine University of California San Francisco, CA United States; ^4^ School of Nursing and Health Professions University of San Francisco San Francisco, CA United States

**Keywords:** SBIRT, mobile app, digital health, training, implementation, alcohol, drug, depression, anxiety

## Abstract

**Background:**

Translation of knowledge and skills from classroom settings to clinical practice is a major challenge in healthcare training, especially for behavioral interventions. For example, screening, brief intervention, and referral to treatment (SBIRT) is a highly-promoted approach to identifying and treating individuals at risk for alcohol or drug problems, yet effective, routine use of SBIRT has lagged.

**Objective:**

The objective of this paper is to describe the development, pilot testing, and trial protocol of a mobile app based on the theory of planned behavior (TPB) to promote SBIRT skill translation and application.

**Methods:**

Intended for use after classroom training occurs, the mobile app has three primary functions designed to increase behavioral intent to deliver SBIRT: (1) review skills (ie, address knowledge and beliefs about SBIRT), (2) apply skills with patients (ie, build confidence and perceived behavioral control), and (3) report performance data (ie, increase accountability and social norms and/or influence). The app includes depression and anxiety screening tools due to high comorbidity with substance use. A randomized controlled trial (RCT) is in progress among health and social service learners (N=200) recruited from 3 universities and 6 different training programs in nursing, social work, internal medicine, psychiatry, and psychology. Participants are randomized to SBIRT classroom instruction alone or classroom instruction plus app access prior to beginning their field placement rotations. TPB-based data are collected via Qualtrics or via the mobile app pre-post and SBIRT utilization, weekly for 10 weeks. Key outcomes include the frequency of and self-reported confidence in delivery of SBIRT.

**Results:**

Beta testing with advanced practice nursing students (N=22) indicated that the app and its associated assessment tools were acceptable and useful. The system usability scale (SUS) mean was 65.8 (n=19), which indicated that the SBIRT app was acceptable but could benefit from improvement. Indeed, modifications were implemented prior to starting the trial. Enrollment of trial participants began in September 2016. Results are expected by December 2017.

**Conclusions:**

This report describes the process of TPB-based app development and testing, and the protocol for a RCT to determine the effectiveness of the app in enhancing skill translation. If effective, this approach could improve SBIRT implementation, fidelity, and clinical outcomes.

## Introduction

The transition from learning clinical skills to sustained changes in health provider behavior is a known problem. Even when evidence-based treatments are available and implementation decisions have been made, workforce development and sustained intervention delivery present formidable challenges [1]. For example, maintaining fidelity to evidence-based treatments (eg, cognitive behavioral therapy [2]), often requires strategies to support ongoing learning such as supervision and coaching [3]. Providers must learn the new skills, practice the skills, build confidence (in themselves and the intervention), and be able to change past practice norms all within an environment that supports such changes. However, these personnel-intensive strategies can be costly and time-consuming, and have limited reach due to resource constraints. Yet with effective strategies to support skill translation [4,5], behavioral healthcare providers can effectively deliver the interventions they are trained to use. Thus, a major challenge for the implementation of evidence-based behavioral practices concerns how to deliver cost-effective support for skill translation in healthcare.

Screening, brief intervention, and referral to treatment (SBIRT) for unhealthy alcohol and drug use is an important example of a widely-trained skill that has fallen short in translation [6]. SBIRT is designed to reach individuals in health or social service settings who use substances at a range of levels, including those who may not yet meet criteria for alcohol or drug use disorders. Components include screening for hazardous drinking and drug use and related problems, delivering brief motivational interviewing-based interventions for patients at low to moderate risk, and providing referrals to addiction specialty care for those with significant problems [7]. Available evidence supports the effectiveness of screening and brief intervention in addressing hazardous drinking within primary care [8-10], although evidence for effectiveness in reducing drug use is weak and trials have been mixed [11-14]. Based on the strength of this literature, national practice guidelines for SBIRT integration into primary care and other health and social service settings have been developed [15,16].

In spite of these practice recommendations and a proliferation of SBIRT training programs, optimal skill translation to direct clinical care remains unrealized. Trainees often demonstrate classroom skill proficiency yet fail to use SBIRT in subsequent clinical placements. Commonly cited barriers to translation include provider attitudes about substance use interventions, problems with knowledge recall at the point-of-care, lack of confidence, inadequate knowledge of referral resources, as well as structural barriers in clinical settings such as limited time and competing medical demands, especially in primary care [17-19]. Studies of SBIRT skill translation and implementation have found a decrease in post-training SBIRT delivery rates over time [20,21], variability in delivery rates across health disciplines [22,23], and low fidelity to screening questions [24]. Fewer than one in six Americans report being asked about or discussing their drinking with a health professional [25], and screening is rarely conducted in US primary care settings [26] outside the Veteran’s Affairs Health System [27]. Similarly, a minority of patients in mental health settings report that providers advise them to reduce hazardous drinking or drug use [28], and a recent meta-analysis demonstrated that fidelity to motivational interviewing by clinicians is often poor [29]. These findings highlight the importance of improving skill translation in real-world health and social service settings.

Digital learning tools have been incorporated into some SBIRT training programs but have not been effectively integrated with clinical care. For example, online training modules sometimes supplement didactic presentations and demonstrations, role play with feedback, and patient encounters [30-32]. With some variability, these digital health training components have been rated as relevant and useful by trainees. Outcome studies have found that such training resulted in increased confidence in SBIRT delivery and more positive attitudes towards patients who use alcohol [33]. Yet digital tools such as online learning models have not supported skill translation over time. To our knowledge, the one SBIRT mobile app that is currently available does not incorporate background materials targeted towards trainees (eg, review of prevalence of substance use and evidence for SBIRT efficacy), nor does it include detailed support in conducting screening, delivering interventions, and treatment referral resources [34]. If designed with skill translation in mind, a point-of-care mobile app with this additional content could help providers apply newly learned SBIRT skills [1].

In other health delivery contexts, mobile apps are gaining acceptance and appear to enhance training. Bullock et al (2015) found that providing a mobile app containing the Dr Companion software with 5 key medical textbooks (the iDoc app) to newly qualified doctors increased access to reference materials and was effective in supporting learning and practice [35]. The use of mobile technology, including medical apps in nursing education, has demonstrated success in improving learning outcomes and learner confidence [36]. Tablet-based patient self-administered alcohol and drug screening [37,38] and intervention [39] can increase efficiency in healthcare settings. Yet prior studies have not examined how mobile apps may enhance SBIRT training and skill translation.

**Figure 1:**
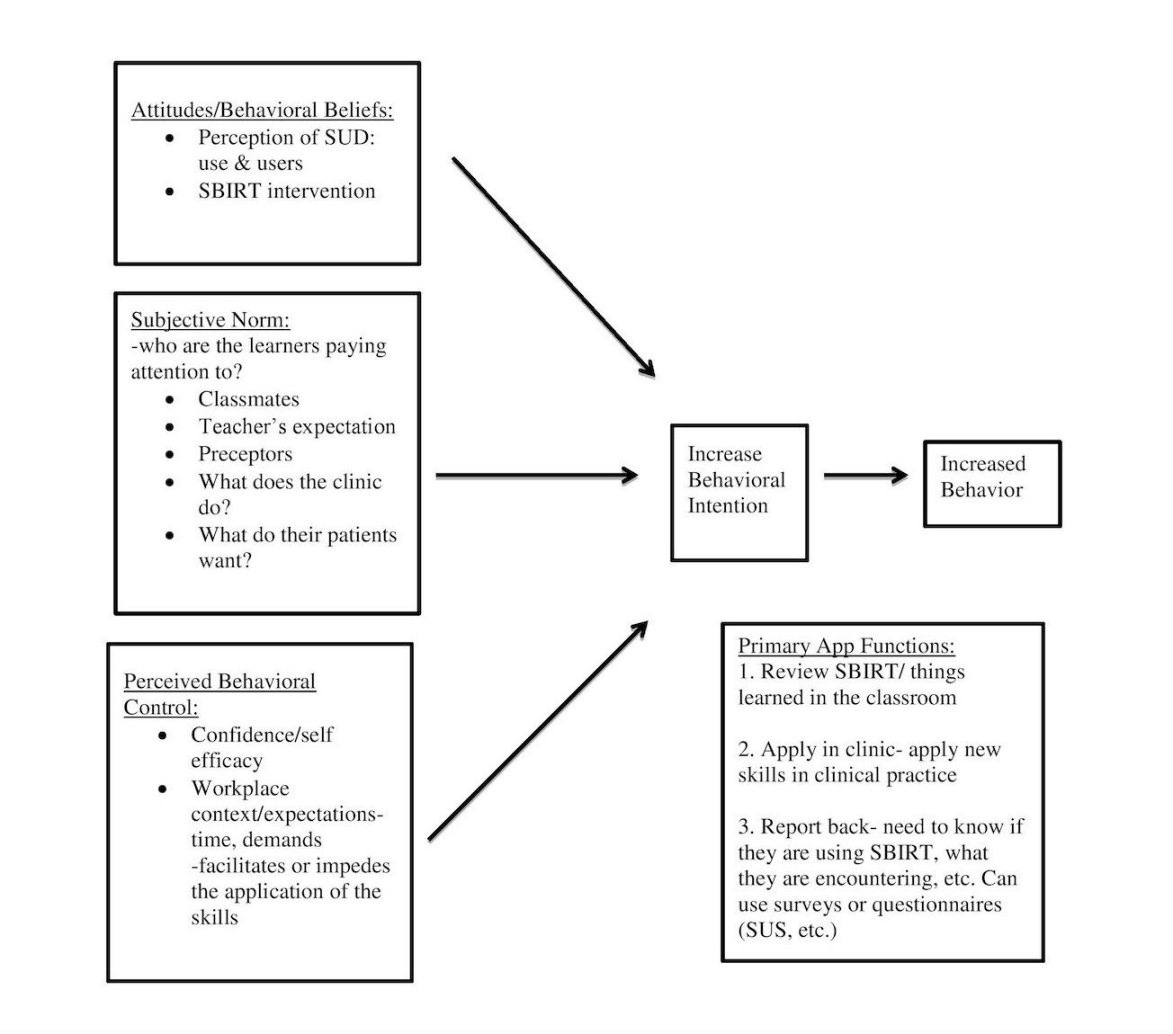
Theory of planned behavior as applied to screening, brief intervention, and referral to treatment (SBIRT) skill translation. Model adapted from Ajzen (1991). SUD: substance use disorder; SUS: system usability scale.

Theoretical models identifying barriers and facilitators regarding learning and skill translation may help guide the development of intervention strategies to enhance skill transfer and implementation. On the level of individual provider (or learner) behavior, the theory of planned behavior (TPB) provides a well-validated, conceptual model that identifies both internal and external key factors that could influence SBIRT skill translation [40]. In the TPB model, behavioral intent (to perform the behavior of interest) is determined by attitudes and/or beliefs about the behavior, perceived social norms, and perceived behavioral control. The TPB is contextualized for SBIRT skill translation in Figure 1, allowing us to assess learners on each of these variables and to provide matched interventions as needed to promote SBIRT usage. For example, we provide information on the extent of substance use and related problems, and SBIRT efficacy to shape attitudes regarding the value of screening and treatment. We provide information on standards of care (eg, that SBIRT has been recommended by the US Preventive Task Force and many health professional bodies) to influence perceived social norms. Moreover, we provide tools to support practicing SBIRT to positively impact both attitudes and perceived behavioral control such as trainees’ confidence in successfully performing SBIRT and integrating it into clinical care.

The aim of this paper is to describe the process of TPB-based mobile alcohol and drug SBIRT app development, beta testing, and protocol for a randomized controlled trial (RCT) comparing health professional learners with access to the app (intervention arm) to learners without access to the app (control arm). We hypothesize that participants in the intervention arm will be more likely to deliver SBIRT in clinical placements than those in the control arm and will be more likely to report intention to deliver SBIRT in the future. We also hypothesize that intervention participants will report more positive beliefs about SBIRT, greater knowledge, and greater perceived control over SBIRT delivery in clinic.

## Methods

### App Development

An interprofessional team worked to develop the TPB-based app. The team was comprised of faculty members with prior research expertise in SBIRT training and implementation and included two clinical psychologists with doctorate degrees, an internal medicine physician, an advanced practice nurse with a doctorate degree, and an experienced project manager. Existing mobile apps were identified to determine needs in the field and to learn about components often emphasized in enhancing behavior change, for example, mobile apps for SBIRT [34], Change Talk for Childhood Obesity [41], and Epocrates [42]. Digital product design principles were reviewed including the creation of a product vision and end goal, character sketches of potential users (“personas”), features of currently available products, and emerging mobile app tools that simplify the user interface and promote app usage [43]. The app’s purpose and vision evolved based on faculty and learner responses to initial designs. As follow-up to a needs assessment questionnaire, faculty members from different training programs were asked about the potential utility of a mobile app to increase learners’ use of SBIRT. Learners identified specific features and content they wanted while using the app during clinic placements. For example, learners wanted point-of-care screening tools and SBIRT frequency of use measures integrated directly into the app.

Based on this initial feedback, the app was designed as a tool for learners to use at their clinic sites that could function as both an SBIRT information resource and as a tool to assist in skills practice and implementation. Content was designed to primarily address alcohol and drug use, but screeners for depression and anxiety, which commonly co-occur with problematic substance use, were added to broaden the scope of the app and to increase its perceived value to both learners and preceptors. A key design principle was to ensure the app fit within the clinical environment and did not disrupt other training or patient care activities. Because of data security concerns and the range of service settings and medical record systems in which trainees could use SBIRT, the app was not designed to connect with local electronic health records and does not record any protected health information. Given the expense and complexity of integrating screening into healthcare records, with which the faculty had prior experience (eg, integration of alcohol screening into electronic health records in Kaiser Permanente Northern California [22]), and the fact that not all learners are placed in settings that have electronic records, we anticipated that keeping the app separate from patient record systems would maximize learner flexibility across various clinical placements and assuage concerns about loss of patient privacy.

Flow diagrams and wireframes (page schematics and screenshots) were drafted to correspond to the key components of the app. Open Health Network app developers [44] were selected as a development partner based on their prior experience in developing mobile apps for healthcare. The wireframes were given to the developers, who provided an initial alpha version. Team members tested the alpha version individually and worked with the developers to continue refining the flow and content for subsequent beta testing.

### Beta Testing

Once the final beta version of the app was developed, our team chose a small cohort of nurse practitioner learners (N=22) at the University of San Francisco to beta test the app for 3 months. Our testers included learners in an advanced practice nursing training program who were enrolled in a clinic placement. Learners completed the questionnaires and processes to be used in the larger RCT. Full TPB-based surveys (see “Pre/Post Assessment Questions”) were repeated at the end of the beta-testing period, followed by a debrief focus group. The team tested and refined the app prior to starting the controlled trial. Beta testing results are described below.

### Randomized Controlled Trial

#### Study Design

**Figure 2:**
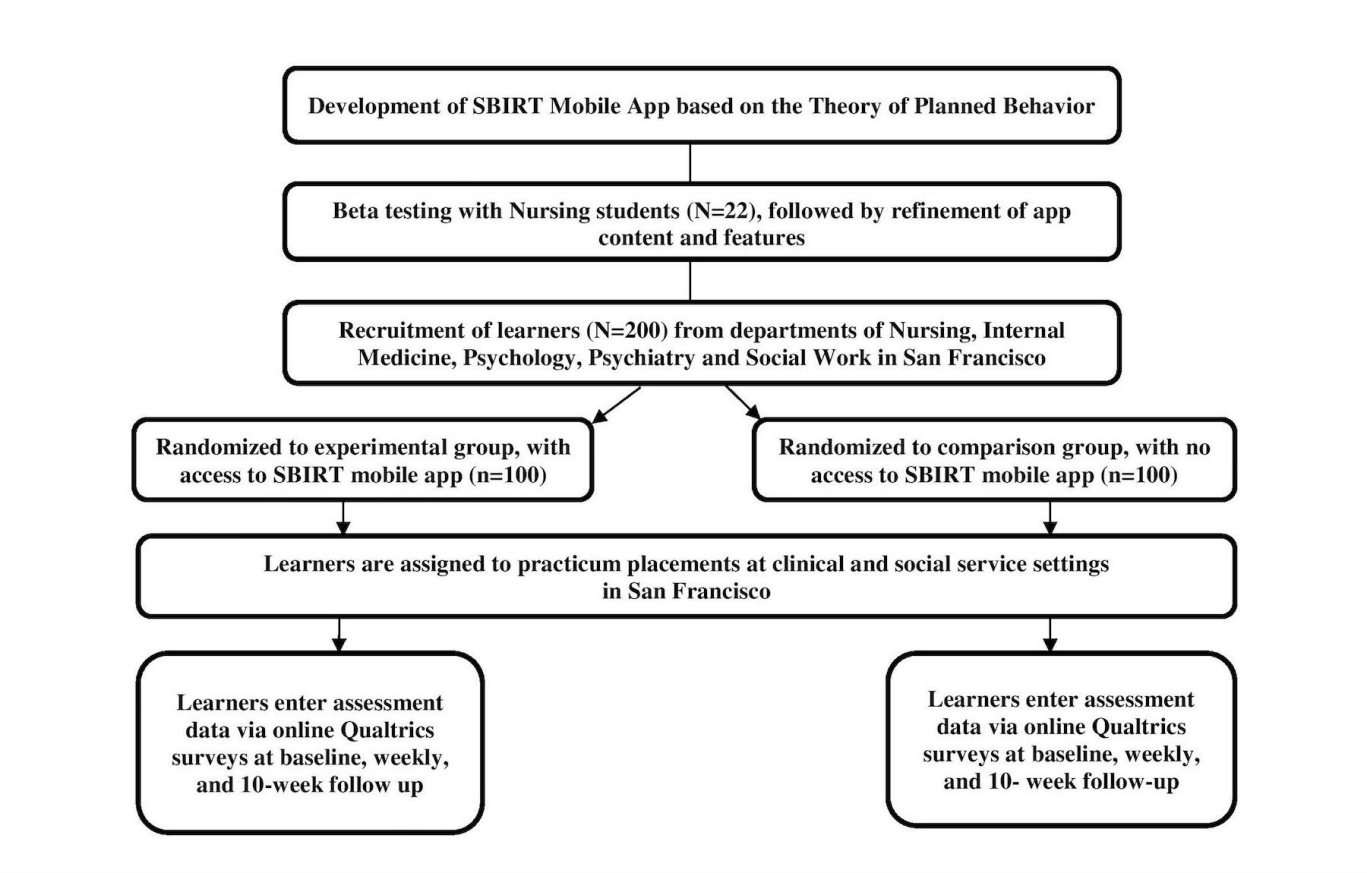
Overall study design for the development and testing of an alcohol and drug screening, brief intervention, and referral to treatment (SBIRT) mobile app to enhance skill translation. Figure adapted from the CONSORT Group.

The study is a RCT of an SBIRT mobile app to facilitate skill translation from classroom to clinical placements among 200 graduate and post-graduate learners (Figure 2). Following SBIRT instruction, participants are enrolled and randomized to the experimental (use of the app) or control (no access to the app) condition. All participants complete self-report measures over the study duration (10 weeks). The trial runs from fall 2016 through spring 2017.

#### Setting

The trial is being carried out at 3 sites, among 6 training programs: San Francisco State University (Social Work and Nursing), University of California, San Francisco (Internal Medicine and Psychiatry), and University of San Francisco (Nursing and Psychology). Institutional review boards at the 3 sites approved all study procedures. The study is not registered with ClinicalTrials.gov because the design does not include a collection of clinical data or patient-level outcomes.

#### Participants

Study participants (trainees, N=200) are adult health professional learners in one of the designated training programs. Participants must have had prior classroom or online training in SBIRT within the past year and may not have previously used an SBIRT app. For learners who have not yet completed classroom-based SBIRT instruction, they are required to complete the following 3 online training modules developed by the research team: (1) Introduction to SBIRT, (2) Screening, and (3) Brief Intervention. The learner must be enrolled in a field placement and is required to have a mobile device to be in the study (Android or iOS). Field placements include a range of private and public healthcare and social service agencies in the San Francisco Bay Area.

#### Recruitment and Randomization

Project faculty identified SBIRT educators at the participating training programs and received permission to recruit students. Students are recruited during scheduled classroom time using a detailed information sheet that specifies expectations, timing, and types of data to be collected. Absent learners are invited to participate via email recruitment. Participants provide informed consent to participate either live in classroom settings or via email.

Standard randomization procedures used in behavioral intervention studies are followed [45] using a variable block size with a 1:1 allocation to the intervention or control arm design. These procedures are carried out by the study project manager. Learners are assigned an identification number that is used for the randomization. A Web-based randomization tool is used to generate group assignments. Randomization is stratified by training program in order to have an even distribution of learners in the intervention and control groups from each program.

#### Intervention Arm

Participants in the intervention group are asked to download the app, use it in their clinical rotations (either on their personal mobile phones or tablets), and complete periodic questionnaires via a Qualtrics link. The learners have the opportunity to use the app as much as they need to review SBIRT, receive guidance on structured steps in SBIRT delivery, and receive tailored recommendations on what they can do to improve. We included modest incentives for app use to maximize our ability to measure the potential effects of app use in the trial.

#### Control Arm

Participants in the control group do not download or use the mobile app in their practicum placements but have access to usual teaching materials and supervisors. Control participants complete periodic questionnaires via a Qualtrics link throughout the study period. Upon completion of the study, they are invited to download and use the app at their discretion.

#### Data Collection

At baseline, all participants answer a TPB-based questionnaire via Qualtrics (control) or directly on the app (intervention). At the end of each week, all participants are asked to respond to a brief Qualtrics survey about how often they used SBIRT either by text message (short message service, SMS) or by email. Upon completion of their clinical rotation, all learners are asked to repeat the original TPB-based questionnaire and to provide general feedback about either the app usage (intervention) or their general satisfaction with SBIRT (control).

#### Incentives

Learners in both groups receive incentives for participating. The incentives are intended to enhance motivation of the learners to use the mobile app and complete study questionnaires. The learners receive Amazon gift cards throughout the study valued at US $20 at baseline, $2.50 for each completed SBIRT usage questionnaire, and $20 at the end of the study for answering the final questionnaire. Maximum payment is US $65 plus participation in a US $50 gift card lottery based on the completion of the SBIRT usage questionnaires.

#### Measures

Data on participants include demographic characteristics, training institution and level of training, type of patients served in clinical placement, and pre-post TPB-based questions to capture the TPB constructs described above (Table 1).

**Table 1:** Measures used in the screening, brief intervention, and referral to treatment (SBIRT) mobile app randomized controlled trial (RCT).

Source	Data elements	Instrument	Timeline
All participants	Demographic characteristics	Self-report	Baseline
All participants	Type of clinical placement and population served	Self-report	Baseline
All participants	SBIRT^a^ attitudes, norms, behavioral control	TPB^b^-based survey (22 items)	Baseline and 10 weeks
All participants	Delivery of SBIRT components during clinical placement	Survey	Weekly
All participants	Satisfaction with the app and usability	SUS^c^ (10 items)	10 weeks
Control participants	Satisfaction with SBIRT	Survey (10 items)	10 weeks

^a^SBIRT: screening, brief intervention, and referral to treatment.

^b^TPB: theory of planned behavior.

^c^SUS: system usability scale.

### Pre-Post Assessment Questions

The team developed a 22-item questionnaire based on the TPB model. Likert-scaled items assess attitudes and beliefs including importance and efficacy of SBIRT, perceived patient willingness to participate in SBIRT, substance use epidemiology and clinical significance, and subjective norms and perceived behavioral control in the clinic setting. Three items assess confidence in the respondents’ ability to screen for alcohol or drug use problems, deliver a brief intervention, and to make referrals. One item assesses intent to perform SBIRT “whenever possible in my clinical/field placement.” All participants complete this questionnaire at baseline and again at 10 weeks. For intervention participants, baseline TPB responses are used to tailor their app experience by making specific recommendations of what the learner might need to review within the app’s library.

The system usability scale (SUS) [46] is a 10-item Likert scale instrument developed to measure aspects of usability including system complexity and need for support and training. It yields a single score ranging from 0 to 100. Intervention group participants complete this measure at follow-up.

### Satisfaction

We developed a 10-item Likert scale questionnaire to measure the experiences of control group participants, as a counterpart to the SUS. Items include barriers and challenges to SBIRT delivery to determine why some participants might not have used SBIRT in the context of their clinical placement. We included the satisfaction questionnaire for control participants only because we want to ensure they have an equivalent number of questions to the intervention participants, and we were concerned about survey burden with our intervention participants.

### Utilization.

At the end of each clinic week, every participant is sent (via email or text) a Qualtrics link asking them to report the total number of patients they have seen in the preceding week. Participants then are asked how many of those patients they screened for alcohol, drug, or tobacco use, how many they did a brief intervention with, and how many they either referred to a specialty substance use treatment clinic or discussed with their field supervisor.

### Intervention

#### Design and Function

**Figure 3:**
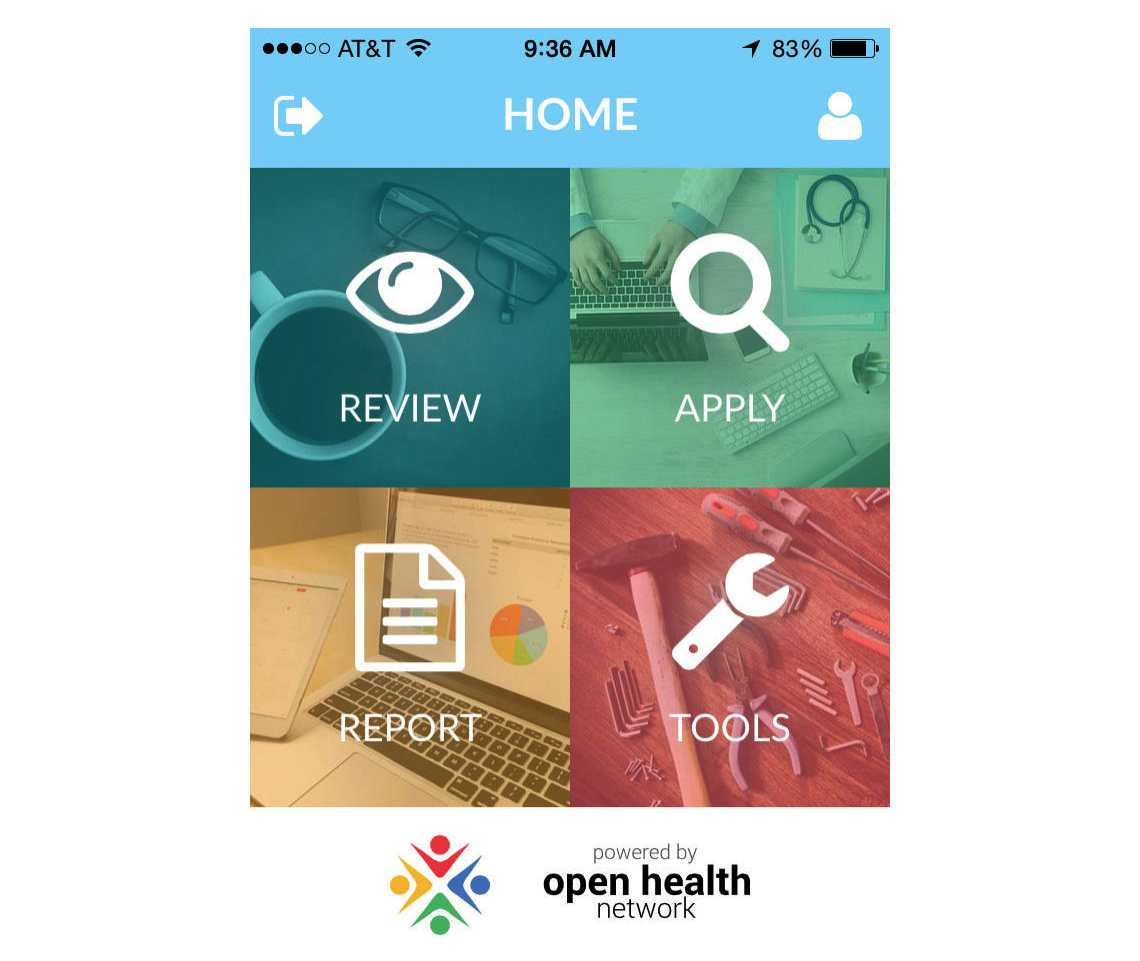
Screen shot of the screening, brief intervention, and referral to treatment (SBIRT) mobile app home screen.

The SBIRT app has the following three primary functions to address TPB concepts: (1) Review SBIRT skills (to help change beliefs and attitudes), (2) Apply SBIRT skills in clinic practice (to help impact attitudes, perceived behavioral control), and (3) Report SBIRT use (to report norms, as well as study outcomes). A fourth component, the Tools section, includes additional reference material and links (Figure 3). After downloading the app, intervention learners create an account and complete the pre-TPB survey. Immediate TPB results are given to the learners along with tailored recommendations on what they should do next. For example, if learners score low on SBIRT knowledge, they are directed to the Review section. A progress checklist in the Tools section reminds them of “homework” they still need to complete. Throughout the study intervention learners are reminded to use the app via the weekly SBIRT usage surveys and periodic text messages promoting app usage.

#### Review

The Review section includes content taught in the classroom (which is also available to learners online), as well as additional material. Subheadings include “Basics” (eg, epidemiology, drugs of abuse, consequences, defining SBIRT), “Screening” (eg, screening questions, sample scripts, and the Diagnostic and Statistical Manual of Mental Disorders, Fifth Edition [DSM-5] definition of substance use disorder [47]), “Brief Intervention” (eg, brief advice, motivational interviewing, harm reduction), “Referral to Treatment” (eg, referral processes, pharmacotherapy), and “Key Resources” (eg, case illustrations, video demonstrations, external links to the National Institute on Alcohol Abuse and Alcoholism, and the National Institute on Drug Abuse).

#### Apply

The Apply section assists learners in using SBIRT while with a patient in clinic placements. This section includes screener instruments, scoring tools, scripts, and step-by-step guidance for delivering a brief intervention or referring a patient to alcohol or drug treatment. For example, the app allows users to specify what they want to screen (eg, alcohol, drugs, depression, anxiety), tailors those questions by gender and age group (18 to 64 versus 65 and up), includes single-question hazardous drinking and drug use screeners, as well as the Alcohol Use Disorders Identification Test (AUDIT) [48], CRAFFT (CRAFFT is a mnemonic acronym of first letters of key words in six screening questions) [49], and Drug Abuse Screening Test (DAST) [50]. Depression screening using the Patient Health Questionnaire (PHQ-2 and PHQ-9) [51] and anxiety screening using the Generalized Anxiety Disorder (GAD-2 and GAD-7) [52] measures are also included due to high comorbidity with substance use and commonalities in intervention approaches. Other subsections include tips for delivering brief interventions, including brief negotiated interviews/motivational interviewing, and suggestions for making referrals (eg, referral processes, lists of local treatment resources, and national treatment locators).

#### Report

The Report section was originally conceptualized as a library of tools for instructors and clinical preceptors to track and evaluate their learners. We initially included a collection of pre-post surveys and weekly SBIRT usage items that intervention participants would complete. In order to standardize the data collection procedures across control and intervention participants, all weekly SBIRT usage surveys were completed via Qualtrics. Intervention participants still completed the pre-post TPB surveys and final satisfaction surveys on the app. However, instructors do not receive reports regarding use of the app or SBIRT by learners.

#### Tools

**Figure 4:**
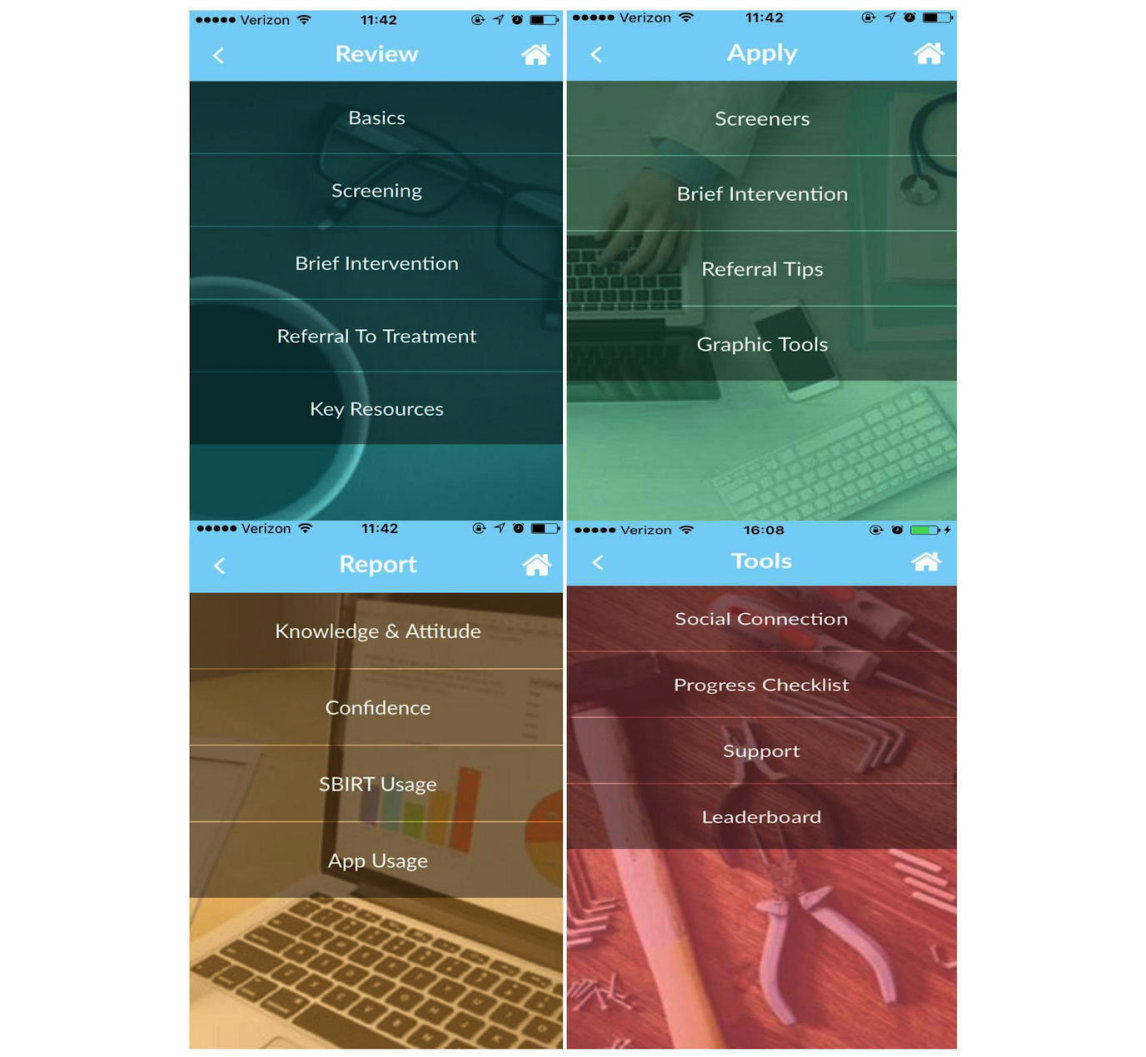
Screenshots of the Review, Apply, Report, and Tools subsections of the screening, brief intervention, and referral to treatment (SBIRT) mobile app.

The Tools section includes social networking, feedback and tracking, and gamification or incentive-building tools. Social networking tools include the “social connection” to send message questions to the study team or other app users. The “Progress Checklist” allows learners to check their progress on which pages they have visited and which pages they still need to review. “Technological Support” is included in this section for those who have technical difficulties and can contact the app developers directly for help. “Leaderboard” is a page on which other learners who are using the app are listed, along with a point system indicating frequency of app use. Leaderboard rankings were tied to lottery tickets and bonus incentives (Figure 4).

### Analyses

Analyses will be conducted using SAS (SAS Institute Inc., 2011). Descriptive statistics, including distributions, means, standard deviations, skewness, and kurtosis will be obtained for all variables. Continuous measures will be tested for normality and homogeneity of variance. If the distribution is normal, Likert-scale responses will be analyzed as continuous scores [53]. Chi-square tests, student *t* tests, and analysis of variance (ANOVA) tests will be used to determine inclusion in multivariate regression models. Bivariate analyses will examine rate of SBIRT delivery in the two arms and comparison of TPB-based measures (eg, beliefs about SBIRT, social norms and influence, and perceived behavioral control). Multivariate analyses (logistic and multiple regression) will examine the impact of these factors on SBIRT delivery.

## Results

### Beta Testing

Initial results focus on beta testing with student learners from the University of San Francisco School of Nursing. Beta testing was completed in summer 2016. The SUS mean was 65.8 (n=19) which indicates that the SBIRT app is acceptable but needs improvements before rolling out to a larger study sample. Debrief participants reported satisfaction with the Apply and Review sections, which included brief intervention scripts, video demonstrations, the level of detail included in the “Referral to Treatment” section, and inclusion of the PHQ-9 (because this is often required in clinic settings to screen for depression). Suggestions for improvement focused on ease of sign-on and reducing the need for navigation (eg, by having multiple scale items appear on a single screen). These formative beta test data were used for app improvement in preparation for the RCT.

### Randomized Controlled Trial

Enrollment of trial participants began in September 2016 and recruitment is ongoing. Trial results are anticipated to be available in late 2017.

## Discussion

### Principal Findings

**Figure 5:**
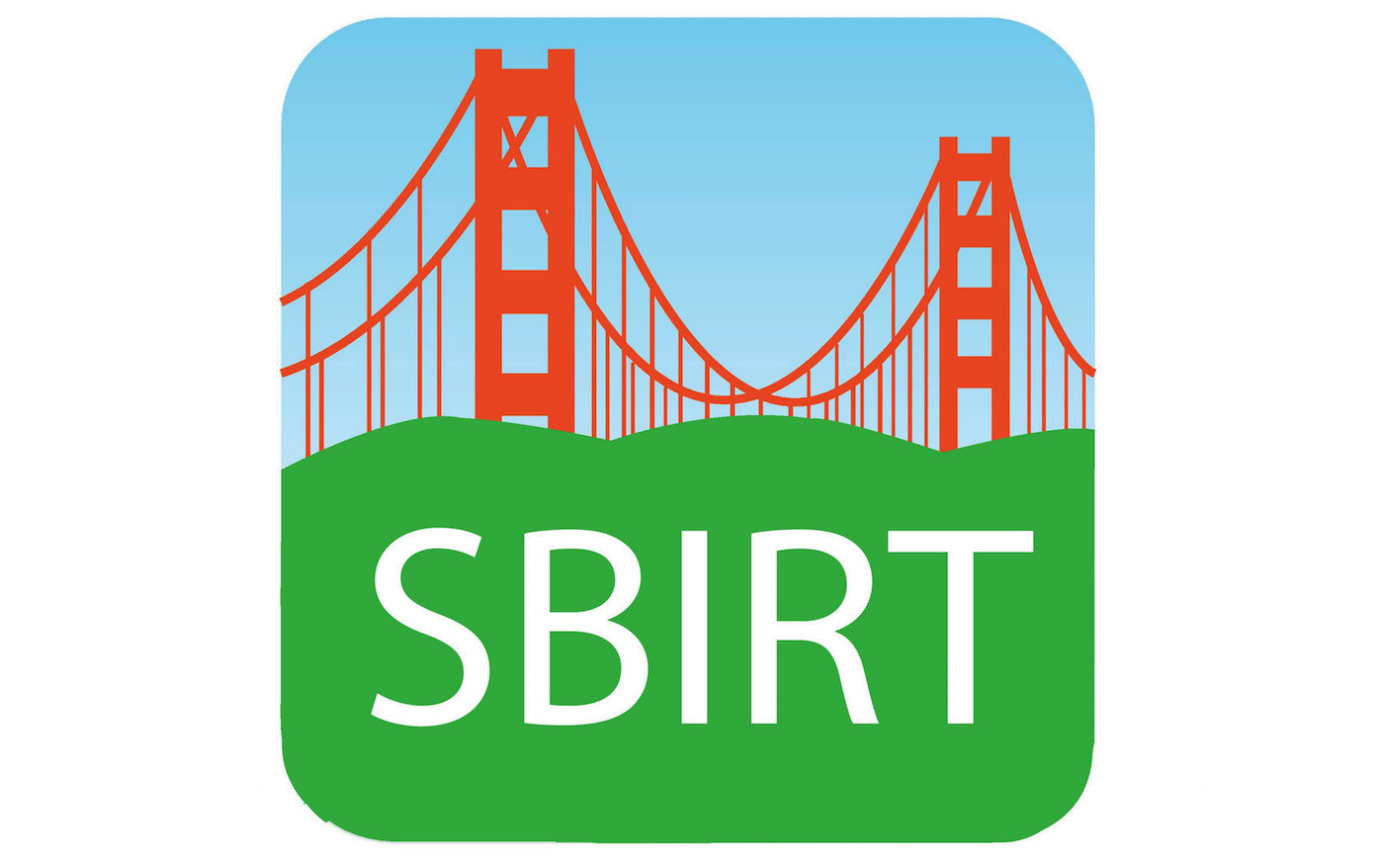
Icon of the screening, brief intervention, and referral to treatment (SBIRT) mobile app.

The study team found that the TBP model was a useful framework for SBIRT mobile app development and that beta testers responded positively overall to the content and features of the app. The app was developed as a tool to promote translation of substance use screening and intervention skills from classroom to clinical settings. Our intent was to assist in workforce development and promote the broader use of evidence-based interventions to reduce alcohol and drug problems among patients in healthcare and social service settings. We used an app to support SBIRT skill translation, embedded in a TPB-based approach to learning, in order to inform the field regarding how mobile app technology may be used to reinforce pedagogy, improve implementation, and enhance patient care. The app, “UCSF OHN SBIRT App,” has been positively reviewed online [54] and is now publicly available for free downloading (iOS only) via the iTunes store [55] (Figure 5). The RCT in process will determine whether the app has a significant impact on SBIRT skill translation, including rates of SBIRT delivery, learner attitudes, and intent to deliver SBIRT.

Based on the evidence and the need for intervention tools usable across settings to reduce alcohol- and drug-related problems, SBIRT instruction in both graduate training programs and continuing education settings for healthcare professionals has been spearheaded by the US Substance Abuse and Mental Health Services Administration, and training opportunities have expanded rapidly over the past 10 years. If efficacy is demonstrated, the mobile app developed by the study team may serve as a useful tool to improve training for healthcare providers and enhance patient care.

This theory-based mobile app serves as a reference guide, a clinical tool, and a data collection instrument. Learners are expected to complete the initial TPB assessment questions before starting their clinical rotations and are then asked to use the app as often as possible during the course of providing direct care. The reporting function frequency of completion is dependent on the structure of the clinical rotation and the needs of the training program and/or preceptors. Although learners’ use of SBIRT is ultimately limited by what their clinical rotation and preceptor allows, this tool may increase the likelihood of effective SBIRT delivery in healthcare and social service settings. This initial presentation describes our mobile app development process, beta testing, and randomized trial methods, which aim to determine the potential impact of this digital tool.

### Limitations

The RCT is conducted in the context of graduate training in the schools in nursing, psychology, social work, and medicine, and may not generalize to other types of professional training or to providers learning SBIRT in the context of continuing education. A limitation of the trial methodology that could impact our study results is the inclusion of incentives within the intervention arm for participants to use the app, which likely would not exist in actual clinical settings. In addition, some clinical settings and supervisors may not be support the use of SBIRT, and this could impact a learner’s ability to use the app. Although use of mobile devices is becoming widespread, limitations in access to technology could impact the reach of this tool [36,56]. The app was not designed to integrate responses to screening measures to electronic health records, which could limit its applicability in some clinical settings. Similarly, issues such as adherence to app usage, appropriate use of technology in the workplace, etiquette, and distraction need to be addressed in future studies [57] to effectively integrate mobile apps into health and social service settings.

### Conclusions

In behavioral health, mobile apps have primarily been directed toward patients, including alcohol and drug use reduction [58-61], smoking cessation [62], management of depression [63], and other mental health conditions [64,65]. Our approach is innovative in that it uses a skill translation theory-based intervention to target care providers and improve service delivery for important behavioral health problems. If effective, the mobile app could be scaled-up to reach a wider clinical audience and may be useful in future work on developing models of SBIRT fidelity and broader approaches to improving skill translation.

## Abbreviations

PHQ: Patient Health Questionnaire
RCT: randomized controlled trial
SBIRT: screening, brief intervention, and referral to treatment
SUS: system usability scale
TPB: theory of planned behavior

## Appendix

Multimedia Appendix 5 CONSORT eHealth checklist V1.6.1.
